# CXCR3^+^ monocytes/macrophages are required for establishment of pulmonary metastases

**DOI:** 10.1038/srep45593

**Published:** 2017-03-30

**Authors:** Kiah L. Butler, Eleanor Clancy-Thompson, David W. Mullins

**Affiliations:** 1 Department of Microbiology and Immunology, Geisel School of Medicine at Dartmouth College, Lebanon, NH 03756; 2 Department of Medical Education, Geisel School of Medicine at Dartmouth College, Hanover, NH 03755, USA.

## Abstract

We present a new foundational role for CXCR3^+^ monocytes/macrophages in the process of tumor engraftment in the lung. CXCR3 is associated with monocytic and lymphocytic infiltration of inflamed or tumor-bearing lung. Although the requirement for tumor-expressed CXCR3 in metastatic engraftment has been demonstrated, the role of monocyte-expressed CXCR3 had not been appreciated. In a murine model of metastatic-like melanoma, engraftment was coordinate with CXCR3^+^ monocyte/macrophage accumulation in the lungs and was sensitive to pharmacologic inhibition of CXCR3 signaling. Tumor engraftment to lung was impaired in CXCR3^−/−^ mice, and transient reconstitution with circulating CXCR3-replete monocytes was sufficient to restore engraftment. These data illustrate the paradoxical pro-tumor role for CXCR3 in lung immunobiology wherein the CXCR3 axis drives both the anti-tumor effector cell chemoattraction and pro-tumor infiltration of the lungs and suggests a potential therapeutic target for lung-tropic metastasizing cancers.

The immune system plays a frustratingly dichotomous role in tumor progression and development. Although host immunity typically mounts an effective anti-tumor effector response during early tumor development, evidence is accumulating that the immune system also aids in the establishment of metastases. Because metastases require: (1) the release of cells from the primary tumor, (2) migration of the cells via circulation or lymphatics, and (3) extravasation at a distal site capable of supporting tumor survival and growth, myeloid cells are fundamentally relevant because they: (1) contribute to the formation of pre-metastatic niches^1^,^2^, (2) recruit and retain cancer cells at the metastatic site^3^,^4^, and (3) promote tumor survival and growth of tumors^5^,^6^. Indeed, depletion of macrophages^3^,^5^, or inhibition of macrophage functional activity by mutation of *CSF-1*^7^, impairs tumor progression and metastasis in murine models. Further, recent work using a model of metastatic-like melanoma has revealed that monocytes/macrophages mediate successful metastasis through binding of the vascular endothelium^4^ and subsequent tethering of tumor cells^8^.

At a molecular level, pro-metastatic macrophages have been characterized as uniquely expressing VEGFR1, CCR2, and CX3CR1, while lacking surface CXCR4 expression^3^,^9^, suggesting that these cells are distinct from proangiogenic macrophages^6^. Kitamura *et al*. have demonstrated that CCR1 and CCR2 participate in monocyte/macrophage recruitment and accumulation in the tumor-challenged lung^5^, but no study has evaluated the relevance of other myeloid cell-expressed chemokine receptors in the process of tumor engraftment. We and others have shown that CXCR3 mediates the migration of lymphocytes and monocytes into inflamed^10^ and tumor-bearing^11^ lung. Of note, tumor-expressed CXCR3 is a key facilitator of tumor progression and metastasis in breast cancer^12^, colon cancer^13^, colorectal cancer^14^, osteosarcoma^15^, and melanoma^16^,^17^. In metastatic breast cancer models, *systemic* pharmacologic inhibition of CXCR3 decreased lung metastasis^12^,^18^, although the relative role of tumor- or myeloid-cell expressed CXCR3 could not be experimentally distinguished with this methodology.

Given that (1) macrophages are required for tumor recruitment and localization to the pre-metastatic niche^3^,^5^,^19^, (2) immune cells are known to localize to lungs in a CXCR3-dependent manner^10^,^11^, and (3) the production of CXCR3 ligands in the lung is induced by introduction of circulating melanoma cells^11^, we hypothesized that CXCR3^+^ monocytes/macrophages may facilitate tumor engraftment in lung. In the present study, we used an established murine model of metastatic-like melanoma to demonstrate a defect in engraftment of lungs in the absence of CXCR3^+^ macrophages. These data highlight a novel role for host CXCR3-expressing monocytes in the establishment of lung metastases, suggesting a potential therapeutic target for lung-tropic metastasizing cancers.

## Results

### Host expression of CXCR3 is required for melanoma engraftment in the lung

**Figure 1:**
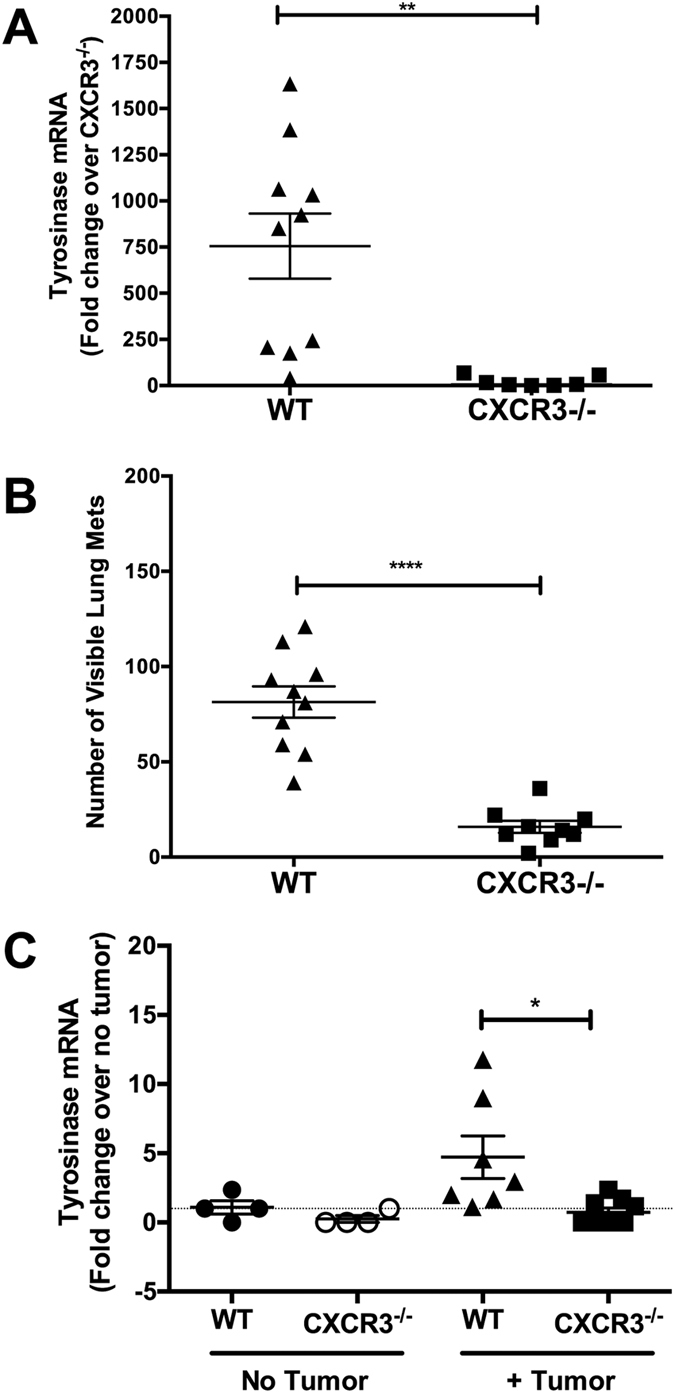
CXCR3 enables the establishment of metastatic-like melanoma in lungs. Mice were intravenously injected with 3 × 10^5^ B16F10 cells. (**A**) Expression of the melanocyte/melanoma-specific gene product *tyrosinase* in lungs in WT and CXCR3^−/−^ mice, assessed 12d post-tumor challenge. (**B**) The number of visible lung metastases in WT and CXCR3^−/−^ mice, assessed 12d post-tumor challenge. (**C**) Expression of *tyrosinase* in lungs of WT and CXCR3^−/−^ mice, with or without tumor, assessed at 24 h post tumor-challenge. In gene expression experiments, qPCR data were normalized to *GAPDH* expression. Data are representative of two independent experiments with similar outcomes, with *n* = 7–10 mice/group in each study (Panels A and B) and *n* = 4–7 mice/group (Panel C). Differences were assessed by T-test as indicated: *p < 0.05; **p < 0.01; ****p < 0.0001.

Metastatic engraftment of tumors, including lung metastases, has been shown to require accumulation of tumor-associated monocytes/macrophages in lung^3^,^5^ and tumor cell expression of CXCR3^12^,^13^,^14^,^15^,^16^,^17^,^18^,^20^,^21^. Although co-localization of monocytes/macrophages and tumor cells is necessary for engraftment, no study has systematically evaluated the role of CXCR3-expressing myeloid cells in tumor engraftment. To address this issue, we assessed metastatic-like tumor growth in WT and CXCR3^−/−^ mice following intravenous injection with B16 melanoma, per established protocols^11^. Twelve days post-challenge, lungs were assessed for expression of the melanocyte/melanoma-specific gene *tyrosinase*^22^ and lung surface metastases were counted. Consistent with our prior observations^11^, lungs from tumor-bearing WT mice expressed high levels of *tyrosinase* (Fig. 1A) and a high number of surface lung metastases (Fig. 1B). In contrast, *tyrosinase* expression and surface metastases were significantly (p < 0.01 and 0.001, respectively) reduced in CXCR3-deficient mice, demonstrating that host cell expression of CXCR3 impacts melanoma engraftment or maintenance in the lungs.

Reduced tumor burden in lungs was demonstrated in CXCR3^−/−^ mice using a long-term orthotopic (4T1 breast cancer) model^12^. However, it remains uncertain whether loss of host CXCR3 impaired initial engraftment or long-term survival of disseminated tumor cells. Therefore, we assessed *tyrosinase* expression in lungs 24 h post-injection as a quantitative measure of melanoma engraftment^23^. Expression of *tyrosinase* was detected in lungs of tumor-challenged WT mice, but *tyrosinase* levels in CXCR3^−/−^ mice was not different than control (no tumor challenge) mice (Fig. 1C). At this time point, there were no observed differences in *tyrosinase* expression in the spleen, regardless of host genotype or tumor injection (not shown), supporting the premise that *tyrosinase* expression in lungs is representative of engraftment. Systemic (Supplemental Fig. 1A) or tumor-restricted (Supplemental Fig. 1B) inhibition of CXCR3 signaling with the pharmacologic antagonist AMG487 reduced melanoma engraftment in the lung, in keeping with prior reports^16^,^20^, demonstrating congruency with our model; interestingly, systemic AMG487 treatment enhanced melanoma engraftment of liver (Supplemental Fig. 1C), suggesting the role of CXCR3 in metastatic engraftment is confined to specific organs, including the lungs. Collectively, these data demonstrate that host expression of CXCR3 is necessary, but not sufficient, to mediate the establishment of melanoma metastases in the lung.

### CXCR3^+^ monocytes/macrophages mediate melanoma engraftment in the lung

**Figure 2:**
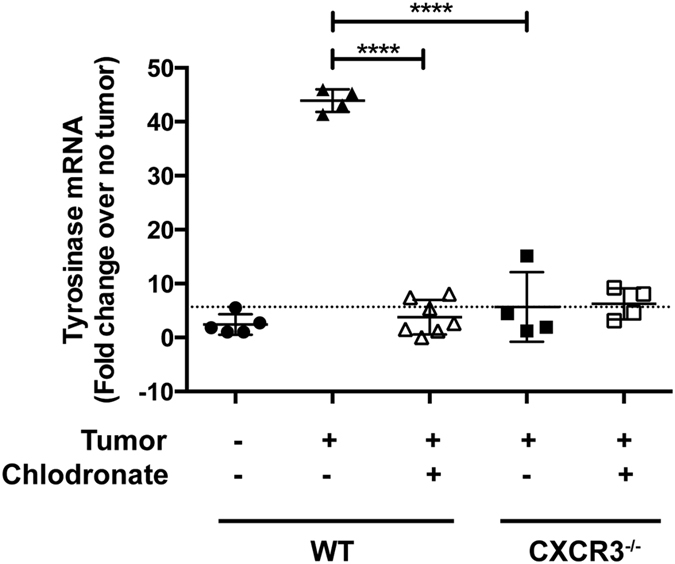
Removal of circulating monocytes ablates melanoma engraftment in the lung. Mice were intravenously injected with 3 × 10^5^ B16F10 cells. Some mice received a macrophage-depleting treatment (chlodronate-containing liposomes) prior to tumor injections. Engraftment was measured by relative expression of *tyrosinase*, normalized to GAPDH, 24 h post-injection of tumor. Data are representative of two independent experiments with similar outcomes, with *n* = 4–7 mice/group in each study. Differences were assessed by T-test as indicated: ****p < 0.0001.

**Figure 3:**
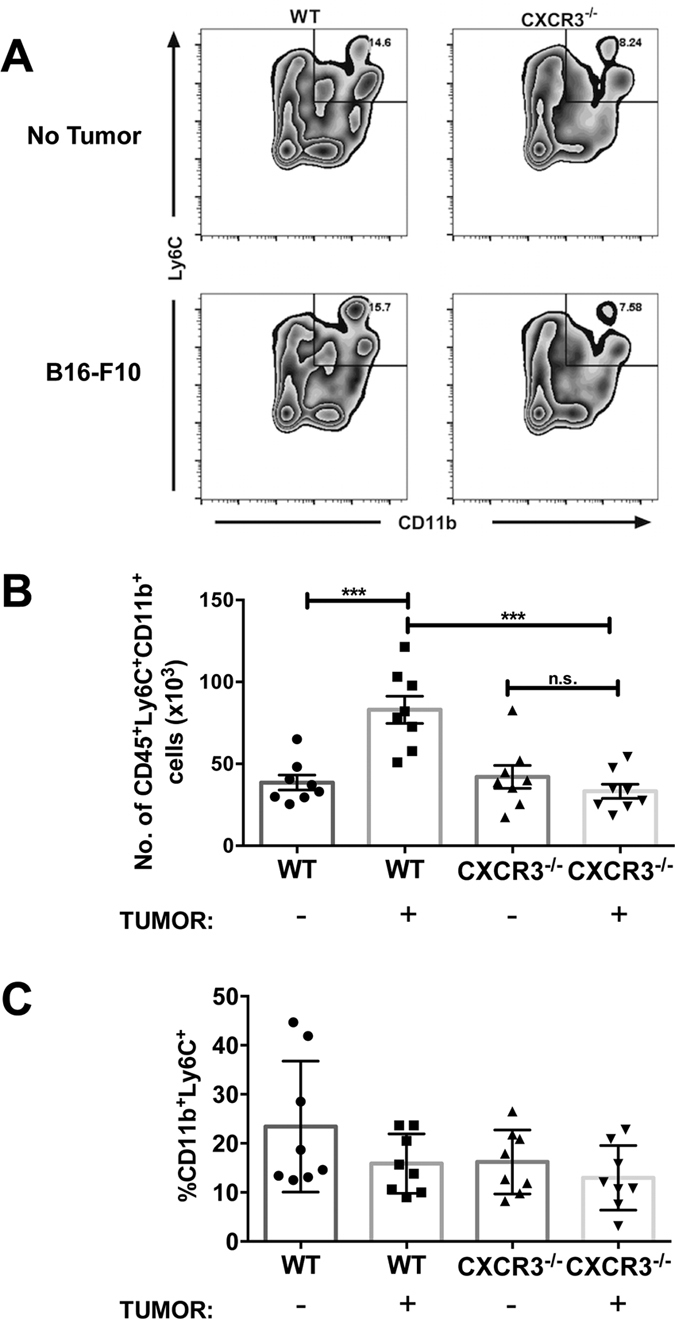
Tumor-induced accumulation of monocytes in lung is compromised in CXCR3^−/−^ mice. Mice were intravenously injected with 3 × 10^5^ B16F10 cells. Twenty-four hours following B16F10 tumor injection, single cell suspensions of lungs were analyzed by flow cytometry. (**A**) Representative staining for circulatory-type monocytes (CD45^+^CD11b^+^Ly6C^+^) in lungs. Gated on viable CD45^+^ events. (**B**) Total numbers of lung monocytes. (**C**) Proportion of lung monocytes. Differences were assessed by T-test as indicated ***p < 0.001.

**Figure 4:**
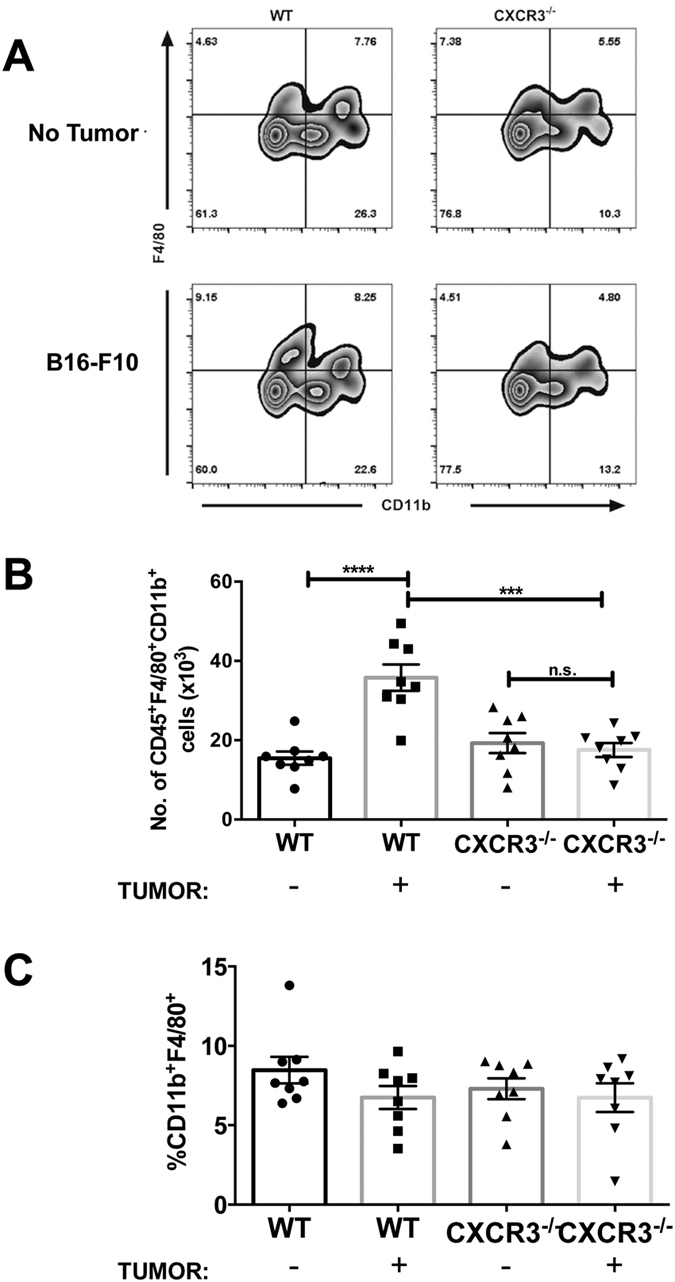
Tumor-induced accumulation of macrophages in lung is compromised in CXCR3^−/−^ mice. Mice were intravenously injected with 3 × 10^5^ B16F10 cells. Twenty-four hours following B16F10 tumor injection, single cell suspensions of lungs were analyzed by flow cytometry. (**A**) Representative staining for macrophages (CD45^+^CD11b^+^ F4/80^+^) in lungs. Gated on viable CD45^+^ events. (**B**) Total numbers of lung macrophages. (**C**) Proportion of lung macrophages. Differences were assessed by T-test as indicated ***p < 0.001; ****p < 0.0001.

Based on our initial data, we hypothesized a pivotal role for hematopoietic expression of CXCR3 for mediating melanoma engraftment in lung. CXCR3 is expressed by monocytes and macrophages^24^,^25^,^26^,^27^ and associated with homing and infiltration of lymphoid and myeloid cells into inflamed lung^10^, which can be replete with CXCR3 ligands^11^. To address whether CXCR3^+^ monocytes/macrophages mediate melanoma engraftment in the lung, we first evaluated melanoma engraftment in WT and CXCR3^−/−^ mice following the depletion of circulating monocytes and macrophages using clodronate liposomes (L-clodronate)^28^ prior to tumor inoculation. Clodronate treatment ablated *tyrosinase* expression in lungs of WT mice (Fig. 2). In keeping with the observation that macrophage-mediated tumor engraftment is associated with macrophage transmigration into lung tissue^4^,^5^, we observed significantly increased numbers of circulatory-type monocytes (CD45^+^CD11b^+^Ly6C^+^, representative staining in Fig. 3A, total cell numbers in Fig. 3B) and macrophages (CD45^+^CD11b^+^F4/80^+^, representative staining in Fig. 4A, total cell numbers in Fig. 4B) in lungs of tumor-challenged WT animals (24 h post-injection), compared with non-tumor controls, although the proportion of monocytes (Fig. 3C) and macrophages (Fig. 4C) in the lungs was unchanged by introduction of tumor. In contrast, there were no significant differences in the numbers or proportions of monocytes (Fig. 3) or macrophages (Fig. 4) in the lungs of tumor-free or tumor-challenged CXCR3^−/−^ mice, consistent with a failure of myeloid cell transmigration without in tumor.

While melanoma engraftment correlated with increased monocyte/macrophage numbers in lungs, the numbers of monocytes in spleen did not differ with introduction of tumor in WT or CXCR3^−/−^ mice (Supplemental Fig. 2A); a modest increase in macrophage numbers in spleen of WT, but not CXCR3^−/−^ mice, was observed with introduction of tumor (Supplemental Fig. 2B).

**Figure 5:**
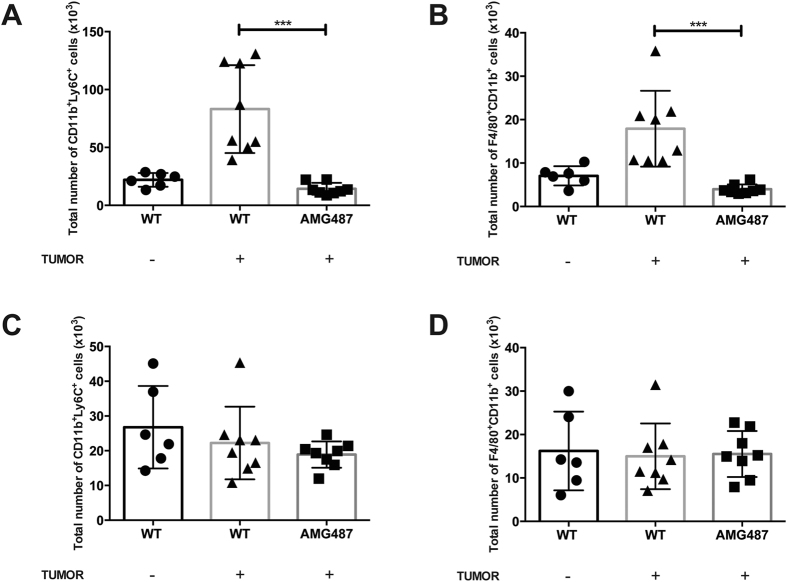
CXCR3 signaling blockade with AMG487 inhibits the tumor-associated accumulation of macrophages or monocytes in lung. AMG487 or vehicle (n = 8 per group) was injected subcutaneously, −1 d and 0 d before B16F10 tumor injection. Mice were intravenously injected with 3 × 10^5^ B16F10 cells or HBSS control. Twenty-four hours later, lungs and spleen were harvested for flow cytometry. F4/80^+^CD11b^+^ cells were assessed in the lung (**A**) and spleen (**B**) and circulating CD11b^+^Ly6C^+^ cells were assessed in the lung (**C**) and spleen (**D**). In all panels, differences were assessed by T-test as indicated: ***p < 0.001.

Systemic blockade of CXCR3 signaling with AMG487 in WT mice abrogated tumor-associated increases in monocyte and macrophage numbers in lungs (Fig. 5A,B, respectively), but not in spleen (Fig. 5C,D). Collectively, these data establish a correlation between tumor engraftment and monocyte/macrophage accumulation in lung, implicating CXCR3^+^ monocytes/macrophages as cellular mediators of engraftment.

**Figure 6:**
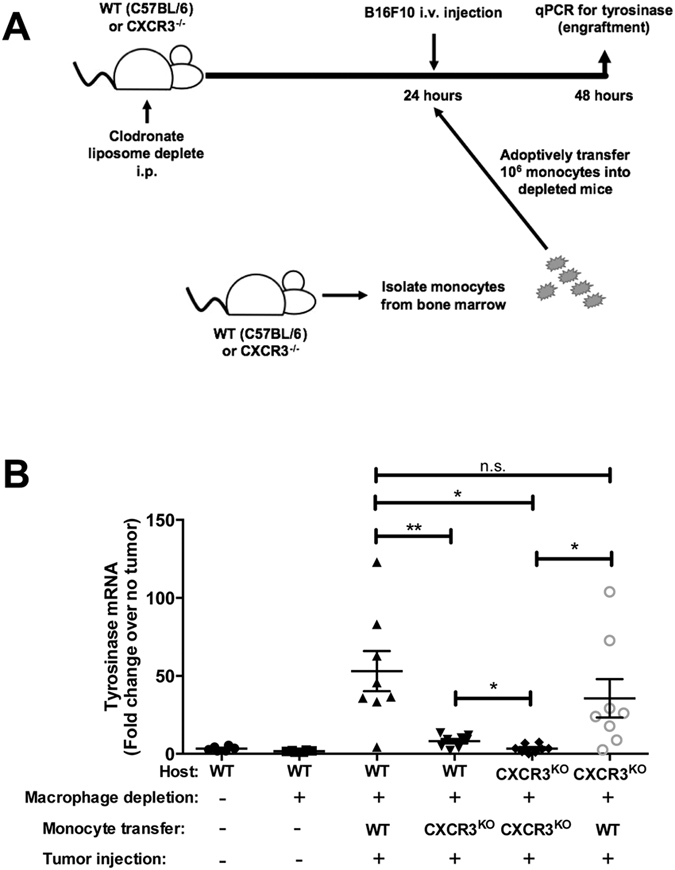
CXCR3-expressing monocytes are required for tumor engraftment in lungs. (**A**) Mice were intravenously injected with 3 × 10^5^ B16F10 cells, then selectively depleted of monocytes/macrophages and reconstituted with WT or CXCR3^−/−^ monocytes. (**B**) *Tyrosinase* expression, measured in the lung 24 h after tumor inoculation. Gene expression data were normalized to GAPDH. Data are representative of two independent experiments with similar outcomes, with *n* = 4–8 mice/group in each study. Differences were assessed by T-test as indicated: *p < 0.05; **p < 0.01.

To confirm the role of CXCR3-expressing macrophages in tumor engraftment in lung, we assessed engraftment in animals depleted of circulating macrophages then selectively reconstituted with CXCR3^WT^ or CXCR3^−/−^ monocytes (schema depicted in Fig. 6A). Tumors efficiently engrafted the lungs of WT mice following macrophage depletion and reconstitution with WT monocytes, whereas reconstitution with CXCR3^−/−^ monocytes failed to restore engraftment (Fig. 6B). Importantly, reconstitution of macrophage-depleted CXCR3^−/−^ hosts with WT, but not CXCR3^−/−^, monocytes allowed intravenously-injected melanomas to engraft lungs (Fig. 6B). This is the first observation of tumor engraftment in CXCR3-deficient hosts and evidence that CXCR3^+^ monocytes are necessary cellular mediators of transmigration for circulating tumor cells to infiltrate the lung compartment.

## Discussion

In 1889, Stephen Paget proposed the “seed and soil” hypothesis, postulating cross-talk between certain cancer cells (the “seed”) and organ microenvironments (the “soil”)^29^ to create tumor-receptive pre-metastatic niches. Recently, the role of the immune system in shaping this niche has been increasingly appreciated^30^. Specifically, macrophages and monocytes have been recognized as having a significant role in attracting, tethering, transmigrating, and supporting metastasizing cancer cells^3^,^4^,^5^. We further refine this work, demonstrating that CXCR3 expression by circulating monocytes is required for efficient engraftment of metastatic-like cancer in the lung. While other chemokine/receptor pathways have been implicated in monocyte-mediated metastasis^5^, the requirement for CXCR3 expression by monocytes themselves has not been previously realized. This role for CXCR3 is distinct from its expression by tumor cells to achieve metastatic engraftment^13^,^16^,^31^, and prior studies using systemic therapies to block CXCR3 signaling have not delineated the relative contributions.

The CXCR3 axis plays an important role in inflammatory^10^ and anti-tumor^11^ processes in lungs, including the induction of CXCR3-cognate chemokine production. Lung injury, including transient ischemia, can rapidly induce CXCL9 and CXCL10 production^32^. Recent work has demonstrated that dual production of the CXCL10 chemokine and CXCR3 receptor by tumor cells contributes to tumor cell metastatic potential and colonization of the lung^33^. Our data are consistent with chemoattraction of circulating CXCR3^+^ monocytes to lungs following injection of tumor cells, which may induce the production of chemokines.

While our study demonstrates the requirement of CXCR3-expressing monocytes for efficient tumor engraftment, the cellular mechanism remains undefined. Monocytes/macrophages mediate chemoattraction^4^ and tethering of tumor cells^6^ in the premetastatic niche, and lung-expressed CXCR3 ligands may signal CXCR3^+^ monocytes to drive one or both of these processes. We and others^4^,^5^ have correlated monocyte accumulation in lung with tumor engraftment, and signaling via CXCR3 may induce or enhance monocyte transmigration. Direct engagement of tumor cells and circulating monocytes/macrophages is a second mechanism that could be exploited by melanoma cells to promote survival in target tissue. VCAM-1 expressed by tumor cells has been shown to engage VLA-4 expressed by circulating leukocytes in breast cancer metastasis in order to promote survival in the lung^8^. Interestingly, engagement of VLA-4 was not shown to play a role in migration (data not shown); however, CXCR3 may induce increased expression of VLA-4 and promote interaction between tumor cells and melanoma cells. On going studies will further elucidate the mechanistic role of monocyte/macrophage-expressed CXCR3 in this system.

These data illustrate the paradoxical role for CXCR3 in lung immunobiology, whereby the CXCR3 axis drives both tumor engraftment and anti-tumor effector cell chemoattraction and infiltration of the lungs. In the case of primary disease or established metastases, production of CXCL9 and CXCL10 correlates with efficient influx of NK and T cells into the tumor microenvironment^11^,^34^, whereas, CXCR3 expression on tumor cells contributes to metastatic potential and colonization of the lung^33^. Thus, we propose that CXCR3 plays varied roles in the immune response to cancer at various stages of tumor dissemination and development, and pan inhibitors of CXCR3 signaling could have pleotropic effects. Thus, we present a new role for CXCR3 in the process of melanoma engraftment. Circulating melanoma cells encountering pulmonary vessels may be too large to pass through and become physically lodged within that tissue. In response, local endothelial cells, and potentially the tumor cell itself, could increase local production of CXCL9 and CXCL10. The gradient established by these cells could act as a signal in circulation and the presence of CXCR3 expression in the host could facilitate recruitment of circulating macrophages and monocytes, which in turn could permit the successful engraftment of melanoma cells in the lung. Our lab is pursuing targeted intervention strategies to dysregulate myeloid cell CXCR3 activity in order to reduce metastatic engraftment and tumor progression in the lungs.

## Materials and Methods

### Cell culture and metastatic-like melanoma model

B16F10 cells were newly obtained from ATCC (CRL-6475), and tumors were established from cryo-preserved stocks that had been passaged less than two times. Cells were cultured in RPMI-1640 (Hyclone, GE Healthcare Life Sciences, Pittsburgh, PA) supplemented with 10% fetal bovine serum (Hyclone) and 25 mM HEPES (Hyclone). Cells were incubated at 37 °C with 5% CO_2_.

Tumors were established in C57BL/6 mice (Jackson Laboratory, Bar Harbor, ME; strain 00664; termed WT mice) or CXCR3^−/−^ mice (Jackson Laboratory strain 005796) by intravenous injection of 3.0 × 10^5^ B16F10 melanoma cells. Animals were maintained at the Geisel School of Medicine’s *Center for Comparative Medicine Vivarium*, and all procedures were approved by the Dartmouth College *Institutional Animal Care and Use Committee* (IACUC, protocol mull.dw.1), and all studies were conducted in accordance with approved protocols.

### AMG487 treatment

AMG487, a pharmacologic inhibitor of CXCR3 signaling, was purchased from Tocris Bioscience (Bristol, United Kingdom). For *in vitro* treatment, AMG487 was prepared as a 10 mmol/L stock solution with DMSO; tumor cells or monocytes were cultured in fresh medium containing 1μmol/L AMG487 for 18 h at 37 °C, then washed and injected. For *in vivo* administration, AMG487 was prepared in a vehicle (hydroxypropyl-β-cyclodextrain [Sigma, St. Louis, MO]) by incubation in a sonicating water bath for 2 h with intermittent vortexing. Distilled water was used to bring the final concentration of AMG487 to 20% of hydroxypropyl-β-cyclodextrain. Mice received 5 mg/kg of AMG487 or vehicle control by subcutaneous injection.

### Gene expression

Total RNA was isolated from perfused lungs using an RNeasy kit (Qiagen, Germantown, MD). Isolated RNA was translated into cDNA using a High Capacity RNA-to-cDNA kit (Applied Biosystems/Thermo-Fisher, Carlsbad, CA). Gene expression was quantified by qPCR using master mix and pre-validated gene-specific TaqMan primers (Applied Biosystems), then analyzed on a StepOne Plus instrument (Applied Biosystems).

### Flow cytometric staining and analyses

Perfused lungs were processed into single cell suspensions using enzymatic digestion (Murine Lung Dissociation kit, Miltenyi Biotec, San Diego, CA) and homogenized using a gentleMacs (Miltenyi) instrument. For surface staining, cells were suspended in FACS buffer (1% bovine serum albumin [BSA, Sigma-Aldrich] in PBS) and incubated with indicated antibodies for 30 min. at 4 °C in the dark. Following three washes in FACS buffer, cells were fixed in 0.5% paraformaldehyde (Sigma-Aldrich) for 20 min. Antibodies specific for murine Ly6C (clone HK1.4), CD45 (clone 30-F11), CD11b (clone M1/70), F4/80 (clone BM8), and CD183 (CXCR3; clone CXCR3-173) were obtained from Biolegend (San Diego, CA). Live/dead fixable violet cell stain was obtained from Thermo-Fisher. Data were acquired with a MacsQuant cytometer (Miltenyi) and analyzed using FlowJo (Ashland, OR) software.

### Isolation of bone marrow monocytes and adoptive transfer

Tibia and femur were isolated from donor WT or CXCR3^−/−^ mice. Monocytes were isolated from bulk bone marrow utilizing the Mouse Monocyte Isolation kit (Miltenyi). Isolated monocyte purity was confirmed at >90% by flow cytometry (gating on CD45^+^CD11b^+^F4/80^neg^Ly6C^+^ cells, not shown). In preparation for the reception of donor monocytes, recipient WT and CXCR3^−/−^ mice were depleted of macrophages and monocytes using clodronate-containing liposomes (chlodronateliposomes.com, Amsterdam, The Netherlands) at 10 μL/g, administered 24 h prior to adoptive transfer^28^. 1.0 × 10^6^ monocytes were injected intravenously; 18 h later, lungs and spleen were collected for analysis.

### Statistics

Statistical analyses were performed using Prism software (GraphPad Software, La Jolla, CA). Data are means ± SEM. Data for experimental groups were compared using the unpaired student t-test.

## Additional Information

**How to cite this article**: Butler, K. L. *et al*. CXCR3^+^ monocytes/macrophages are required for establishment of pulmonary metastases. *Sci. Rep.* **7**, 45593; doi: 10.1038/srep45593 (2017).

**Publisher's note:** Springer Nature remains neutral with regard to jurisdictional claims in published maps and institutional affiliations.

## Supplementary Material

Supplemental Figures
