# Supplementary material for: CXCR3+ monocytes/macrophages are required for establishment of pulmonary metastases
Source: Sci Rep. 2017 Mar 30;7:45593. doi: 10.1038/srep45593 (PMC5372355; doi:10.1038/srep45593)
Supplement: Supplemental Figures [file srep45593-s1.pdf]

Supplemental Information

CXCR3$^+$ monocytes/macrophages are required for establishment of pulmonary metastases.

Kiah L. Butler[1,3], Eleanor Clancy-Thompson[1,3], and David W. Mullins[1,2]

[1]Department of Microbiology and Immunology, Geisel School of Medicine at Dartmouth College, Lebanon, NH 03756, and [2]Department of Medical Education, Geisel School of Medicine at Dartmouth College, Hanover, NH 03755.

[3]KLB and EC-T made equal first-author contributions.

Supplemental Figure 1

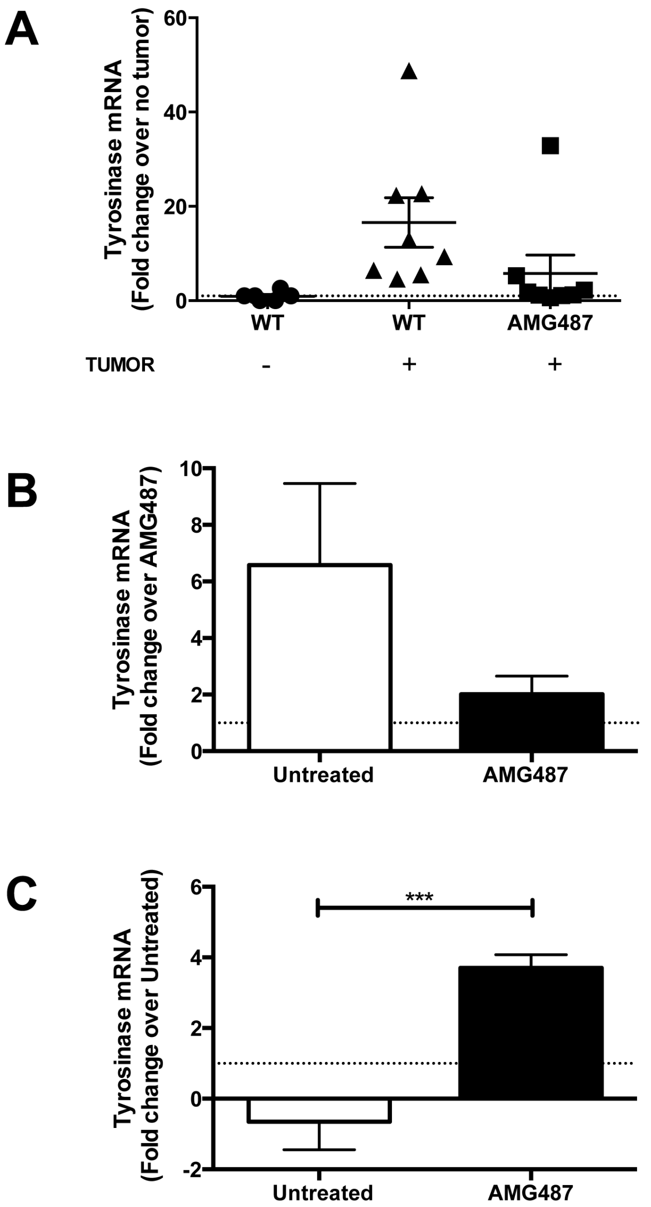

Supplementary Figure 1. Blocking melanoma expression of CXCR3 decreases metastasis to the lungs but increases metastasis to the liver. (A) Mice were intravenously (i.v.) injected with $3x10^5$ B16F10 cells. Engraftment was measured by qPCR for tyrosinase expression was assessed at 24 hours after tumor injection, without or with systemic AMG487 treated mice (B-C) B16F10 melanoma cells were pre-treated with AMG487 for 18 h to block CXCR3 signaling. Mice were intravenously (i.v.) injected with $3x10^5$ B16F10 cells. Twenty-four hours later, tumor burden was assessed in (B) lung and (C) liver by qPCR for tyrosinase and normalized to GAPDH. Differences were assessed by T-test as indicated: *, $p<0.05$; **, $p<0.01$; ***, $p<0.001$.

Supplemental Figure 2

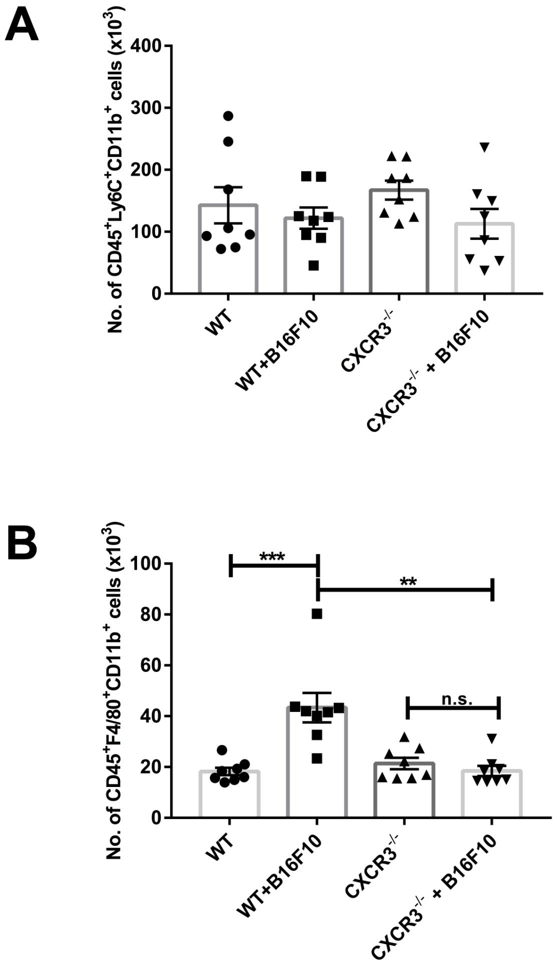

Supplementary Figure 2. CXCR3 expression decreases macrophages in circulation. Mice were intravenously (i.v.) injected with $3x10^5$ B16F10 cells. (A) Total cell numbers of monocytes ($CD45^+CD11b^+Ly6C^+$) isolated from the spleen. (B) Total cell numbers of macrophages ($CD45^+CD11b^+$ $F4/80^+$) isolated from the spleen. Differences were assessed by T-test as indicated: **, p<0.01; ***, p<0.001.
